# mTOR signalling in the nucleus accumbens shell is critical for augmented effect of TFF3 on behavioural response to cocaine

**DOI:** 10.1038/srep27895

**Published:** 2016-06-10

**Authors:** Yi-Xiao Luo, Hua Han, Juan Shao, Yuan Gao, Xi Yin, Wei-Li Zhu, Ying Han, Hai-Shui Shi

**Affiliations:** 1 Department of Pharmacology, Medical School of Hunan Normal University, Changsha 410013, China; 2 Department of gynecology and obstetrics, Hebei General Hospital, Shijiazhuang 050051, China; 3 Department of Senile Disease, the Third Hospital of Hebei Medical University, Shijiazhuang 050000, China; 4 Department of Biochemistry and Molecular Biology, College of basic medicine, Hebei Medical University, Shijiazhuang 050017, China; 5 Department of Functional region of Diagnosis, Hebei Medical University Fourth Hospital, Hebei Medical University, Shijiazhuang 050011, China; 6 National Institute on Drug Dependence, Peking University, Beijing 100191, China

## Abstract

Neuropeptides play important roles in modulating the rewarding value of abused drugs. Trefoil factor 3 (TFF3) was recently reported to modulate withdrawal syndrome of morphine, but the effects of TFF3 on the cocaine-induced behavioral changes are still elusive. In the present study, cocaine-induced hyperlocomotion and conditioned place preference (CPP) rat paradigms were provided to investigate the role of TFF3 in the reward response to cocaine. High-performance liquid chromatography (HPLC) analysis was used to analyse the dopamine concentration. The results showed that systemic TFF3 administration (0.1 mg/kg i.p.) significantly augmented cocaine- induced hyperlocomotion and CPP formation, without any effects on locomotor activity and aversive or rewarding effects per se. TFF3 significantly augmented the increment of the dopamine concentration in the NAc and the activity of the mTOR signalling pathway induced by acute cocaine exposure (10 mg/kg, i.p.) in the NAc shell, but not the core. The Intra-NAc shell infusion of rapamycin blocked TFF3-induced hyperactivity in cocaine-treatment rats. These findings indicated that TFF3 could potentiate behavioural response to cocaine, which may be associated with regulating dopamine concentration. Furthermore, the findings indicated that mTOR signalling pathway in the NAc shell is important for TFF3-induced enhancement on the cocaine-induced behavioral changes.

Drug addiction is a major society public health problem^1^. The rewarding effects of abused drugs are critical for development of drug addiction^2^. Exposure to cocaine and other psychostimulants, causes enduring neuroadaptations within interconnected dopaminergic, GABAergic and glutamatergic projections among the nucleus accumbens (NAc), ventral tegmental area (VTA) and prefrontal cortex (PFC)^3^,^4^,^5^. Activity-dependent gene expression in the related brain regions has been revealed to be an important factor in the reward response and the subsequent neuroadaptations induced by abused drugs^6^,^7^,^8^. Cocaine abuse induces alterations of gene expression by modulating both transcriptional and translational levels, including the rate of mRNA/protein stability and degradation^9^,^10^.

The trefoil factor family (TFFs) peptides are major secretory products of mucin–producing cells and can be synthesized in the brain^11^. Three TFF peptides have been characterized in mammals, including humans: TFF1 (formerly designated pS2), TFF2 (formerly hSP), and TFF3 (also designated hP1.B/hITF). Most previous investigations have focused on the functions of TFF3 in maintenance of mucosal integrity and the promotion of repair as well as in gastrointestinal cancers^12^,^13^. TFF-deficient mice represented an impaired capacity for mucosal recovery after colonic injury even after exposure to antineoplastic agents and total body radiation^14^,^15^. Further evidence from clinical studies showed that a recombinant human TFF3 (rhTFF3) oral spray formulation was safe and effective for the treatment of chemotherapy-related oral mucositis in patients with colorectal cancer^16^.

In the central nervous system (CNS), TFF3 is mainly expressed in neuron and oligodendroglia of the hypothalamus, amygdala, hippocampus and prefrontal cortex (PFC)^11^,^17^,^18^. However, the role of TFF3 in the CNS is still not well understood. It has been found that lack of TFF3 resulted in hearing impairment and accelerated presbyacusis, while another earlier study showed no obvious neural abnormalities in TFF3 knockout mice^15^,^19^. Considering the existence of TFFs in the CNS, they should have specific effects during physical and even pathological processes. The first study reported that an intra-basolateral amygdala (BLA) infusion of recombinant human TFF3 (rhTFF3) regulated anxiety-like behaviour in a dose-dependent manner^20^. We previously found that acute systemic TFF3 administration (0.1 and 0.5 mg/kg, i.p.) enhanced learning and retention of novel object recognition memory and exerted antidepressant-like effects in rodents^21^,^22^. More recently, we reported that systemic TFF3 treatment attenuates naloxone-precipitated withdrawal symptoms via the regulation of glucocorticoid secretion and neuronal activation in the PFC in morphine-dependent mice, indicating that TFF3 potentially has regulatory roles in drug addiction^23^.

Although several potential TFFs-binding proteins have been reported, the cell surface receptor of the TFFs is still unknown. TFF3 can act on epidermal growth factor receptors (EGFR) to activates several downstream signalling pathways, including the mitogen-activated protein kinase (MAPK), phosphatidylinositol 3-kinase (PI3K)/AKT mTOR pathways^24^. Mammalian target of rapamycin (mTOR), an evolutionarily conserved kinase, regulates the translation and transcription of targeted genes via the activation of the downstream molecules, such as the phosphorylation of P70S6 kinase (P70S6K) and S6 ribosomal protein (rpS6). A series of studies have shown that the mTOR signalling pathway is critical for the abused drug induced neuroadaptations and behavioural alterations in both rodents and humans^25^,^26^,^27^. In our previous studies, we found that exposure to cocaine-related cue increased p70s6k and rps6 phosphorylation in the NAc core, but not shell and that intra-NAc core, but not the shell, administration of rapamycin attenuated cue-induced reinstatement of cocaine seeking behaviour, indicating a differential role of the mTOR signalling pathway in NAc sub-regions in drug addiction^26^,^28^.Taking together, TFF3 may have effects on drug addiction through activating the downstream targeted molecular mTOR signalling pathway.

Based on previous findings, the present study aimed to investigate the potential role of TFF3 in cocaine-induced locomotor activity and cocaine-induced conditioned place preference and the underlying mechanisms using cocaine-induced conditioned place preference (CPP) and hyperlocomotion rats paradigms.

## Materials and Methods

### Subjects

Male Sprague Dawley rats (weighing 240–260 g on arrival) were obtained from the Laboratory Animal Center at the Peking University Health Science Center and housed in groups of five under controlled temperature (23 ± 2 °C) and humidity (50 ± 5%). These rats were kept on a 12-h light/dark cycle with access to food and water *ad libitum*, and they weighed approximately 300–320 g when the experiments began. All of the animal procedures were performed in accordance with the National Institutes of Health Guide for the Care and Use of Laboratory Animals and were approved by the Committee of Animal Use and Protection of Hebei Medical University .

### Drugs

Cocaine hydrochloride (Qinghai Pharmaceuticals, Xining, China) was dissolved in sterile saline and injected before the CPP training sessions or the locomotor activity test. Recombinant human TFF3 (purity 98%; Beijing Yong Kang Jia Xin Biotechnology Co., Ltd. Beijing, China) was dissolved in sterile saline. The mTOR inhibitor rapamycin (Sigma, St Louis, MO) was dissolved in 5% dimethylsulfoxide (DMSO)^26^,^29^. The drug concentrations were adjusted to an appropriate injection volume of 1 ml/kg bodyweight for cocaine and TFF3 administration or 0.5 μl/side for intra-NAc core or shell infusion of rapamycin.

### Intracranial surgery and injections

Rats (weighing 300–320 g) were anesthetized with sodium pentobarbital (60 mg/kg, i.p.). Guide cannulae (23 gauge; Plastics One) were implanted bilaterally 1 mm above the NAc core or shell. The cannulae were placed at a 16° angle toward the midline to avoid penetration of the lateral ventricle. The following NAc core coordinates were used: anteroposterior, +1.5 mm; lateral, ±3.8 mm; and dorsoventral, −6.2 mm. The following NAc shell coordinates were used: anteroposterior, +1.8 mm; lateral, ±3.2 mm; and dorsoventral, −6.6 mm^28^. The cannulae were anchored to the skull with stainless steel screws and dental cement. A stainless steel stylet blocker was inserted into each cannula to maintain patency and to prevent infection. All rats were allowed to recover for 5–7 d after surgery.

Rapamycin was infused bilaterally into the NAc core or shell with Hamilton syringes connected to 30-gauge injectors (Plastics One). A total volume of 0.5 μl was infused into each side over 1 min, and the injector was kept in place for an additional 1 min to allow for diffusion. Considering the minor impact on the reward effect of cocaine, a 5 ug/side dose of rapamycin was chosen, which was adjusted from previous reports^28^.

### Histology

**Figure 1:**
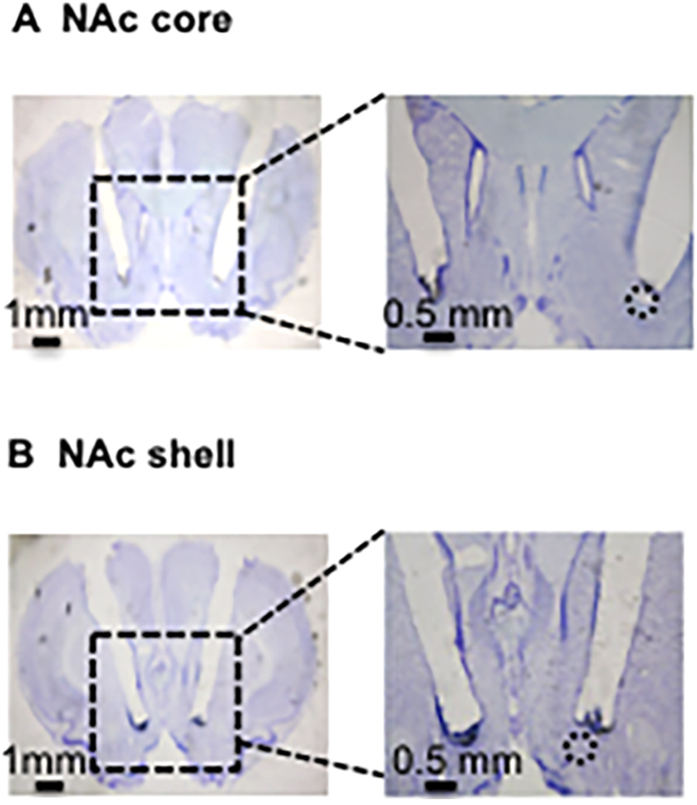
Representative cannula placements in the NAc core and shell.

Because the NAc cores or shells of the experimental group were dissected for further western blot or HPLC analyses, histology slides were obtained from naïve rats that were micro-injected with 5% DMSO in the NAc shell or core. After micro-injection, the rats were anesthetized with sodium pentobarbital (100 mg/kg, i.p.) and transcardially perfused with paraformaldehyde. Cannula placements were assessed by Nissl staining with a 40-μm thickness under a light microscope^28^. The locations of representative cannula tips are shown in Fig. 1.

### Tissue sample preparation and western blot assays

The samples were treated according to our previous studies^30^. Immediately after the locomotion test, the rats were decapitated. The brains were quickly extracted, frozen in −60 °C N-hexane, and transferred to a −80 °C freezer. In a −20 °C environment, bilateral tissue punches (12 or 16 gauge) of the NAc core or shell were taken from 1-mm thick coronal sections ~2.52 mm from the bregma^28^. Protein samples were prepared according to the protocol of radioimmunoprecipitation assay (RIPA) kits (Beyotime Biotechnology, Jiangsu China). All of the above procedures were performed under low temperature (0–4 °C). The protein concentrations of all of the samples were determined using the bicinchoninic acid assay (Beyotime Biotechnology, Jiangsu China), and the samples were further diluted by RIPA lysis buffer to equalize the protein concentrations.

For western blot analysis, equal amounts of total protein (30 ug) for each sample were loaded into 8% or 10% sodium dodecyl sulphate-polyacrylamide gels for electrophoresis. The primary antibodies used were as follows: anti-phospho mTOR (1: 1000, Ser2448, #2971S; Cell Signaling Technology, MA, USA), anti-mTOR (1: 2000, #2972; Cell Signaling Technology, MA, USA), anti-phospho-rpS6 (1: 500, Ser235/236, #2211S; Cell Signaling Technology, MA, USA), anti-rpS6 (1: 1000, 5G10, #2217; Cell Signaling Technology, MA, USA), and horseradish peroxidase-conjugated secondary antibody (1: 5000, SC-2678, SC-2354, Santa Cruz Biotechnology, CA, USA). The Quantity One 4.0.3 software (Bio-Rad, Hercules, CA, USA) was used to quantify band intensities.

### High-performance liquid chromatography (HPLC) analysis

All of the chromatographic procedures were performed as described previously^31^. Similar to the method mentioned above, the rats were decapitated immediately after the locomotion test, and the brains were quickly extracted. Bilateral tissues of the NAc were then taken using 16-gauge punches under low temperature (0–4 °C). The samples were collected in Eppendorf tubes that contained 10 μl of 0.1 M perchloric acid, kept on ice during the collection period and then stored at −80 °C until analysed.

The quantification of the dopamine concentration in the samples was performed using HPLC with a Phenomenex, C18, 150*4.60 m column (Phenomenex, Torrance, CA) and Coularray II 5600A electrochemical detector with a glassy carbon electrode^32^. The mobile phase consisted of 0.76 M NaH_2_PO_4_•H_2_O, 0.5 mM EDTA, 1.2 mM 1-octane sulfonic acid, and 5% acetonitrile perfused using a pump at a flow rate of 0.6 ml/min. The chromatographic data were recorded on a computer, and the peak heights of dopamine in the samples (10 μl) were compared with standards (different concentrations) using chromatography-reporting software (BAS, West Lafayette, IN, USA).

### Locomotor activity test

The locomotor activity test (LAT) procedure was modified from previous studies^33^,^34^ and performed with an automated video tracking system (Dig Behav-LM4, Shanghai Jiliang Software Technology Co. Ltd, Shanghai China) containing eight identical clear Plexiglas chambers (40 × 40 × 65 cm). A monochrome video camera was mounted at the top of each chamber. All of the chambers were connected to a computer that recorded locomotion. The video files (stored on the computer) were analysed using Dig Behav analysis software^35^. Before the beginning of LAT, all of the rats allowed to have 5 minutes habituation phase in the test chamber. Locomotor activity is expressed as the total distance travelled during a 1-h test session.

### Conditioned place preference

The apparatus for CPP training and testing was the same as previously described. To investigate the potential role of TFF3 in the reward response to cocaine, the CPP procedure was adjusted from our previous reports^36^,^37^.

To determine their baseline preferences, rats were initially placed in the middle chamber with the doors removed for 15 min (pre-conditioning test, Pre-T). A computer measured the time spent in the designated saline- or cocaine-paired chambers during the 15-min session through interruptions of infrared beams. Most rats spent approximately one-third of the time in each chamber (data not shown). Approximately 5% of the total rats were discarded because of a strong unconditioned preference toward one chamber (>540 s). Conditioning was performed using an unbiased, balanced protocol.

On the subsequent conditioning days, each rat was trained for 2 consecutive days with alternating injections of cocaine (10 mg/kg, i.p.) and saline (1 ml/kg, i.p.) with a 12-hour interval using an adapted protocol based on our previous studies to avoid the ceiling effects of the rewarding effects^38^,^39^. The injection doses of TFF3 were chosen based on our previous studies. TFF3 (saline, 0.01, 0.1 mg/kg, i.p.) was administered 30 minutes prior to cocaine or saline injection, after which the rats were confined to the corresponding conditioning chambers for 45 min before being returned to their home cages. Considering the potential impact of TFF3 on the cocaine reward effects, a relatively low cocaine dose (5 mg/kg, i.p.) was used in the CPP conditioning processes. The next day, after 2 days conditioning, all of the rats were tested for expression of CPP (post-conditioning test, Post-T) under conditions identical to those described in the pre-conditioning test. The CPP score was defined as the time spent in the cocaine-paired chamber minus the time spent in the cocaine-unpaired (saline-paired) chamber. TFF3 CPP was performed similar to the cocaine CPP, except that the cocaine injections were substituted by TFF3 injections (0.01 and 0.1 mg/kg i.p.).

## Specific Experiments

### Experiment 1: Effects of TFF3 administration on cocaine-induced hyperlocomotion

**Figure 2:**
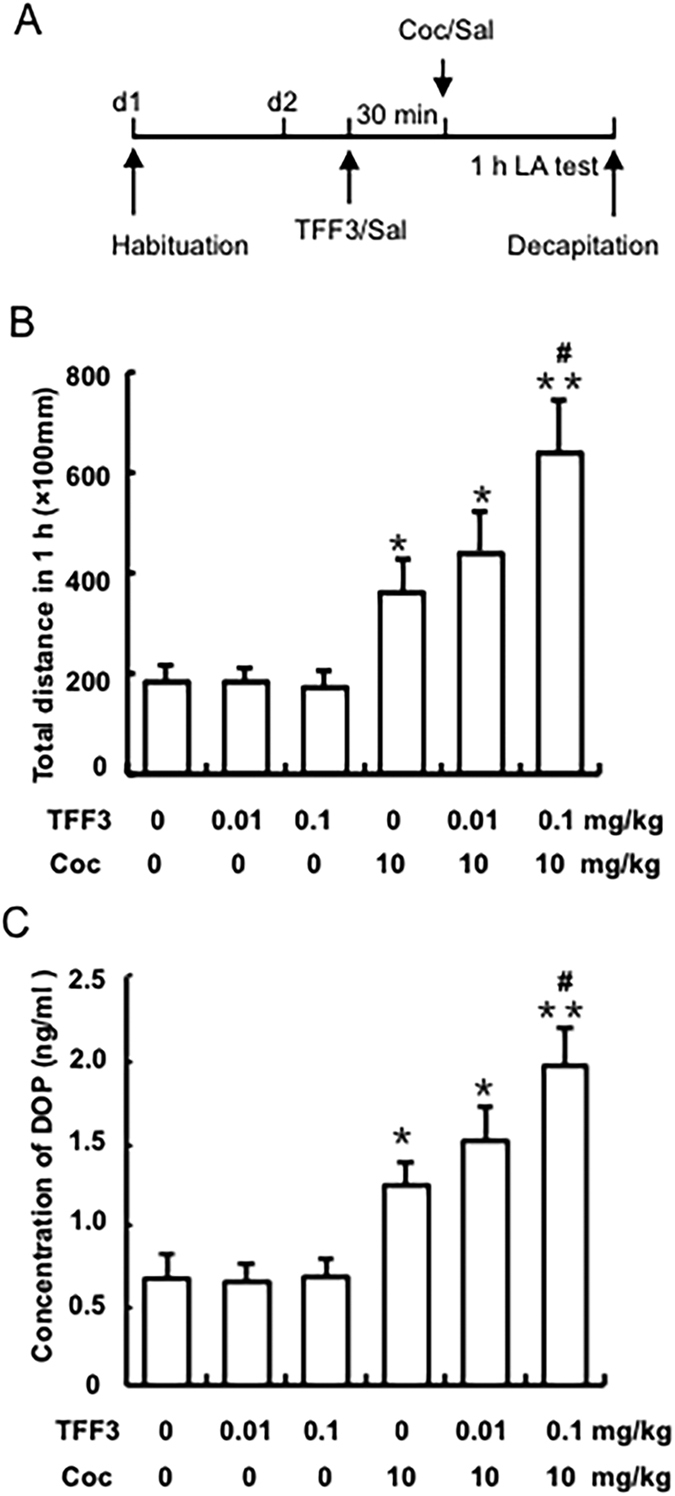
TFF3 administration potentiated the increment of the cocaine-induced hyperlocomotion activity and dopamine concentration in the NAc. (**A**) Behavioural procedure. (**B**) TFF3 administration (0.1 mg/kg, i.p.) 30 min before cocaine injection significantly augmented cocaine-induced hyperlocomotion. (**C**) TFF3 administration (0.1 mg/kg, i.p.) 30 min before cocaine injection significantly promoted the cocaine-induced increase in dopamine concentration in the NAc. Data are expressed as the mean ± SEM. *p < 0.05, **p < 0.01 compared with “saline + saline” group; ^#^*p* < 0.05 compared with “saline + cocaine” group. n = 6–12 per group.

To determine the effect of TFF3 on cocaine-induced locomotor activity. Rats (n = 6–10 per group) were pre-treated with a dose of TFF3 (saline, 0.01, or 0.1 mg/kg, i.p.), and then, 30 min later, they received an i.p. injection of either saline or 10 mg/kg cocaine. All rats received a 1-h locomotion activity test immediately after cocaine/saline injection. Immediately after the 1-h locomotion activity test, the rats were decapitated and tissue from the NAc region was isolated for the detection of dopamine concentration by HPLC (see Fig. 2A).

### Experiment 2: Effects of TFF3 administration on mTOR signalling pathway activity in NAc in cocaine-treated rats

**Figure 3:**
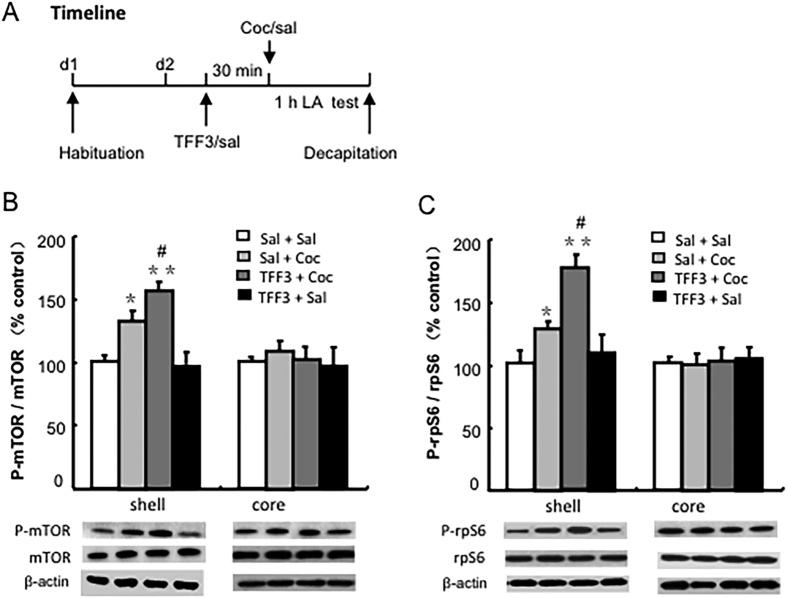
TFF3 administration enhanced the cocaine-induced increase in mTOR signalling pathway activity in the NAc shell. (**A**) Behavioural procedure. (**B**) The ratios of p-mTOR/mTOR and (**C**) p-rpS6/rpS6 were increased in the NAc shell, but without any accompanying change in the NAc core, and the “saline + saline” group was used as the control. Data are expressed as the mean ± SEM. *p < 0.05, **p < 0.01 compared with “saline + saline” group; ^#^*p* < 0.05 compared with “saline + cocaine” group. n=5–7 per group.

To assess the effect of TFF3 on mTOR signalling activity in the NAc region after cocaine treatment, another four groups of rats (6–8 per group) were pre-treated with a dose of TFF3 (saline or 0.1 mg/kg, i.p.) and 30 min later received an injection of either saline (1 ml/kg, i.p.) or cocaine (10 mg/kg, i.p.). 5 minutes after cocaine injection, the rats received locomotion activity test for 1 hour. All rats were decapitated immediately after the locomotor activity test and the total proteins from the NAc shell and core regions were extracted for subsequent determination of p-mTOR and p-rpS6 phosphorylation, respectively (see Fig. 3A).

### Experiment 3: Effects of mTOR signalling pathway inhibition in the NAc core or shell on TFF3-induced hyperlocomotion in cocaine-treated rats

**Figure 4:**
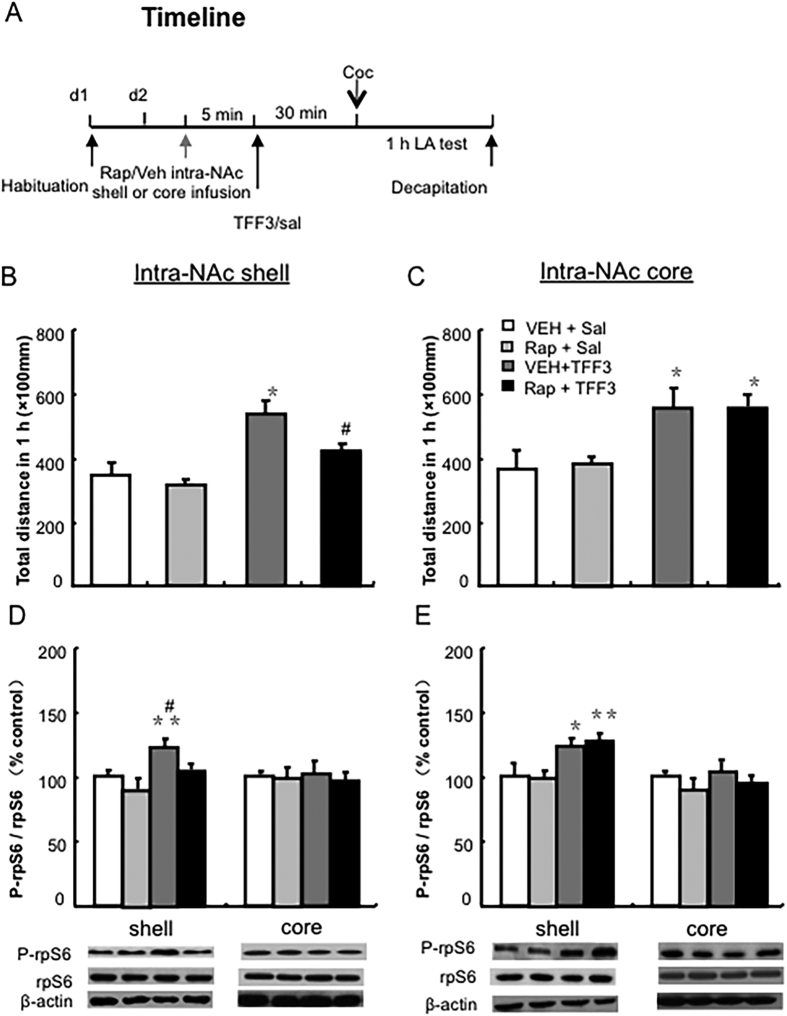
Inhibition of mTOR signalling in the NAc shell blocked the effect of TFF3 on cocaine-induced hyperlocomotion. (**A**) Behavioural procedure. (**B**) Micro-injection of rapamycin into the NAc shell, (**C**) but not the core, prior to TFF3 administration blocked the increment of hyperlocomotion activity induced by TFF3 in cocaine-treated rats. Percentage of p-rpS6/rpS6 after the intra-NAc shell (**D**) or core (**E**) infusion of rapamycin prior to TFF3 administration NAc shell and core, and the “vehicle + saline” group was used as the control. Rap: rapamycin. Data are expressed as the mean ± SEM., **p < 0.01, compared with “vehicle + saline” group; ^#^*p* < 0.05 compared with “vehicle + TFF3” group. n = 6–11 per group.

To determine whether the regulatory effect of TFF3 on hyperlocomotion induced by cocaine is mediated by mTOR signalling in the NAc, rats (n = 14–20 per group) were pre-treated with a dose of TFF3 (saline or 0.1 mg/kg, i.p.) following intra-NAc core or shell infusion with rapamycin (vehicle or 5 ug/side), respectively. Thirty minutes after infusion, all rats received cocaine (10 mg/kg i.p.) followed by locomotion activity test for 1 hour. They were then decapitated to determine the activity of mTOR signalling in the NAc shell or core region (see Fig. 4A).

### Experiment 4. Effects of TFF3 administration on the cocaine-induced conditioned place preference (CPP)

**Figure 5:**
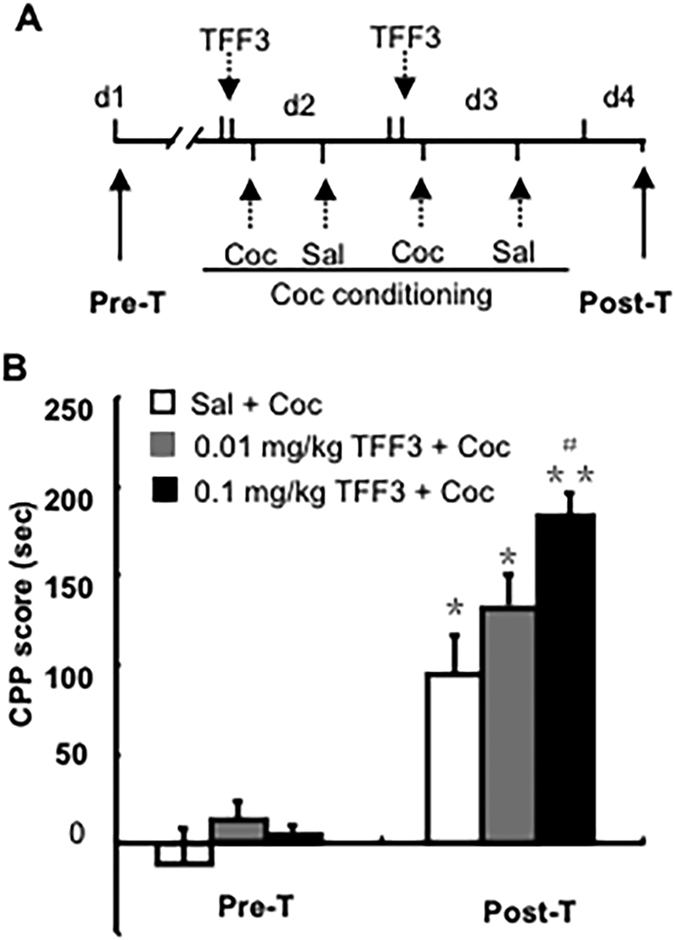
TFF3 administration augmented cocaine-induced conditioned place preference. (**A**) Behavioural procedure. (**B**) TFF3 administration (0.1 mg/kg, i.p.) 30 min prior to cocaine injection during conditioning sessions significantly increased the cocaine-induced CPP. Data are expressed as the mean ± SEM. *p < 0.05, **p < 0.01 compared with the corresponding group in the Pre-T, ^#^*p* < 0.05 compared with “saline + cocaine” group. n = 8–11 per group.

The conditioned place preference (CPP) is a typical paradigm used to evaluate drug reward effect. The CPP rats model is used to assess the effect of TFF3 on the cocaine-induced conditioned place preference. Rats were pre-treated with a dose of TFF3 (saline, 0.01, or 0.1 mg/kg, i.p.). Thirty minutes later they received an injection of either saline (1 ml/k g, i.p.)or cocaine (5 mg/kg, i.p.) followed by an adjusted CPP conditioning procedure (see Fig. 5A).

### Experiment 5: Whether TFF3 administration has reward or aversive effects per se

**Figure 6:**
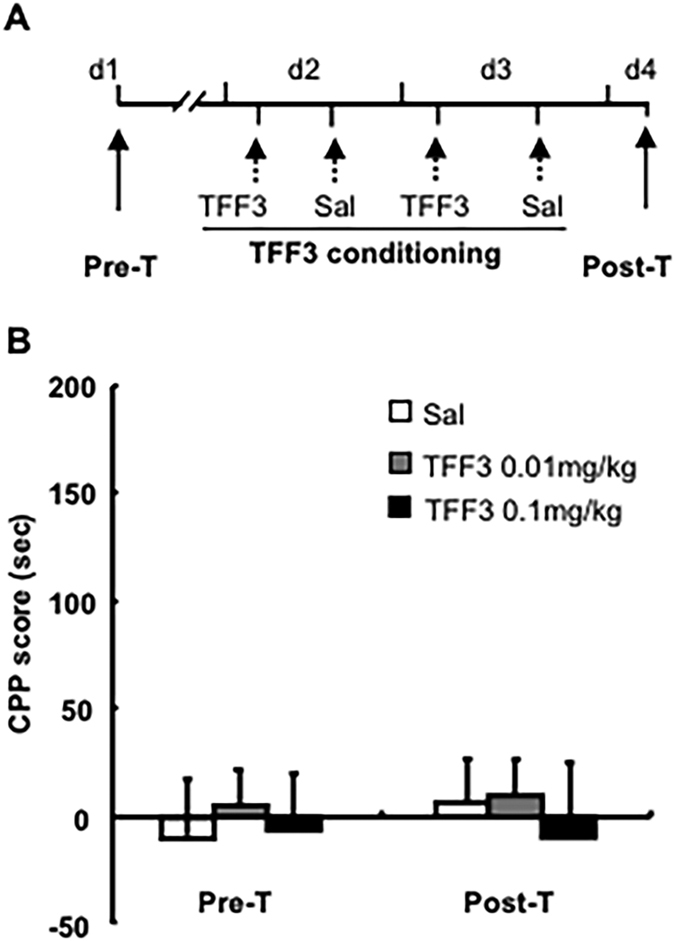
TFF3 had neither aversive nor rewarding effects per se. (**A**) Behavioural procedure. (**B**) TFF3 administration could not induce the formation of CPP. Data are expressed as the mean ± SEM. n = 6–8 per group.

To exclude the aversive or rewarding effects of TFF3 itself, another 3 groups of rats were used for this experiment. Rats were treated with a dose of TFF3 (saline, 0.01, 0.1 mg/kg, i.p.) instead of cocaine and were then trained according to the similar procedure of cocaine CPP conditioning in experiment 4 (Fig. 6A).

### Data analysis

SPSS version 21.0 was used for data analysis, and the data are expressed as the mean ± SEM. Data from the locomotor activity test, HPLC, and western blot were analysed using two-way analysis of variance (ANOVA). Data from the CPP were analysed using repeated measures ANOVA (rm-ANOVA) with TFF3 treatment as the between-subjects factor (saline, TFF3 treatment) and test condition (Pre-T, Post-T) as the within-subjects factor. The Tukey’s test was used for significant effects of *post hoc analysis* (see Results for details). *P*-values < 0.05 were considered statistically significant.

## Results

### TFF3 administration significantly promoted cocaine-induced hyperlocomotion

We firstly tested the potential regulatory effects of TFF3 on cocaine-induced hyperlocomotion. Two-way ANOVA with cocaine treatment (saline and 10 mg/kg) and TFF3 (saline, 0.01 and 0.1 mg/kg) as between-subjects factors, revealed significant main effects for cocaine [F(1, 51) = 25.462, p < 0.01, Fig. 2B] on locomotor activity. Post-hoc test showed that cocaine (10 mg/kg i.p.) administration induced a significant increase in locomotor activity compared to the saline group (p < 0.05, Fig. 2B), and 0.1 mg/kg dose of TFF3 significantly potentiated the cocaine-induced hyperactivity (p < 0.01, Fig. 2B). TFF3 administration alone at either 0.01 nor 0.1 mg/kg dose had no effects on locomotor activity compared to the saline group (all p > 0.05, Fig. 2B). These results suggest that systemic TFF3 administration potentiated cocaine-induced locomotor activity.

To unravel the possible neural mechanisms underlying the effects of TFF3 on cocaine-induced locomotor activity, HPLC was used to detect the dopamine concentration in the NAc region. Two-way ANOVA with cocaine treatment (saline and 10 mg/kg) and TFF3 (saline, 0.01 and 0.1 mg/kg) as between-subjects factors, revealed significant main effects for cocaine on the dopamine concentration in the NAc region [F(1, 33) = 45.243, p < 0.01, Fig. 2C]. Post-hoc test showed that cocaine administration significantly increased the dopamine concentration in the NAc region (p < 0.05, Fig. 2C), which was further augmented by TFF3 at 0.1 mg/kg dose (p < 0.01, Fig. 2C). TFF3 administration (0.01 or 0.1 mg/kg) alone had no effects on dopamine concentration in the NAc compared to the saline group (all p > 0.05). The results indicate that the effects of TFF3 on locomotor activity might be associated with increased concentration of dopamine within the NAc.

### TFF3 significantly enhanced the activation of the mTOR signalling pathway in the NAc shell region of cocaine-treated rats

To further investigate the molecular mechanism underlying the regulatory effect of TFF3 on the cocaine-induced locomotor activity, the activity of the mTOR signalling pathway in the NAc shell and core was assessed by detecting the ratios of phosphorylated mTOR (p-mTOR/mTOR) and the phosphorylated ribosomal protein S6 (p-rpS6)/rpS6, respectively. One-way ANOVA revealed a significant difference in mTOR signalling activity in the NAc shell between groups, as detected by the ratio of phosphorylated p-mTOR and mTOR [F(3, 23) = 20.497, p < 0.01, Fig. 3B] and the ratio of phosphorylated p-rpS6 and rpS6 [F(3, 23) = 44.863, p < 0.001, Fig. 3C]. Post hoc analyses indicated that cocaine (10 mg/kg, i.p.) significantly increased the phosphorylation of p-mTOR and mTOR (p < 0.05, Fig. 3B) and the ratio of p-rpS6 and rpS6 (p < 0.05, Fig. 3C). TFF3 (0.1 mg/kg, i.p.) significantly potentiated the cocaine-induced increment in the phosphorylation of p-mTOR and mTOR (p < 0.01, Fig. 3B) and the ratio of p-rpS6 and rpS6 (p < 0.01, Fig. 3C). However, for the activity of mTOR signalling in the NAc core region, there was no significant difference observed among groups assessed by the phosphorylation of p-mTOR and mTOR [F(3, 19) = 0.631, p > 0.05, Fig. 3B] and phosphorylation of p-rpS6 and rpS6 [F(3, 19) = 0.458, p > 0.05, Fig. 3C].

### Inhibition of mTOR activity in the NAc shell blocked the effect of TFF3 on cocaine-induced hyperlocomotion

To investigate whether the regulatory effect of TFF3 on cocaine-induced hyperlocomotion is mediated by the mTOR signalling pathway in the NAc, rapamycin was microinjected into the NAc shell or core 5 minutes before TFF3 administration. For rats with an intra-shell infusion of rapamycin, the behavioural results showed a significant difference between groups [F(3, 32) = 12.525, p < 0.001, Fig. 4B]. Post-hoc analysis revealed that the intra-NAc shell infusion of rapamycin blocked the augmenting effects of TFF3 on cocaine-induced hyperlocomotion activity (p < 0.05, Fig. 4B). Accordingly, the western blot results showed a significant difference between groups for a ratio of p-rpS6/rpS6 in the NAc shell [F(3, 19) = 3.124, p < 0.001, Fig. 4D], but not for a ratio of p-rpS6/rpS6 in the NAc core [F(3, 21) = 15.080, p > 0.05, Fig. 4D]. Post hoc analysis showed that a TFF3-induced augmented activity of mTOR signalling in the NAc shell, but not in the NAc core, was significantly inhibited by the intra-NAc shell infusion of rapamycin (p < 0.05 Fig. 4D).

For rats with an intra-NAc core infusion of rapamycin, the behavioural results showed a significant difference between groups [F(3, 23) = 20.497, p < 0.01, Fig. 4C]. The intra-NAc core infusion of rapamycin could not block the augmenting effects of TFF3 on cocaine-induced hyperlocomotion activity (p > 0.05, Fig. 4C). Accordingly, the western blot results showed a significant between-group difference for ratio of p-rpS6/rpS6 in the NAc shell [F(3, 17) = 2.197., p < 0.01, Fig. 4E], but not in the ratio of p-rpS6/rpS6 in the NAc core [F(3, 17) = 11.007, p > 0.05, Fig. 4E]. Post hoc analysis indicated that the intra-NAc core infusion of rapamycin could not block the augmented activity of mTOR signalling in the NAc shell (p > 0.05, Fig. 4E). Altogether, these results indicate that mTOR signalling in the NAc shell, but not he NAc core, mediated the regulatory effects of TFF3 on cocaine-induced locomotor activity.

### TFF3 administration potentiated cocaine-induced conditioned place preference

The conditioned place preference (CPP) paradigm was used to further investigate the regulatory effects of TFF3 on the reward response to cocaine (Fig. 5A). Rm-ANOVA with the test condition (Pre-T and Post-T) as the within-subjects factor and TFF3 treatment (saline, 0.01, and 0.1 mg/kg) as the between-subjects factor revealed a significant effect of TFF3 treatment and test condition and an interaction between the TFF3 treatment × test condition [F(2, 24) = 9.797, p < 0.01; F(1, 24) = 116.219, p < 0.01; F(2, 24) = 3.283, p < 0.05, respectively]. Further analysis showed that all of the groups of rats acquired the cocaine-induced CPP and that TFF3 treatment at the dose of 0.1 mg/kg significantly increased the cocaine-induced conditioned place preference (p < 0.05, Fig. 5B).

### TFF3 had no aversive and rewarding effects per se

To exclude the possibility of TFF3 exerting rewarding or aversive effects itself, TFF3-induced CPP was assessed using the similar adapted procedure (Fig. 6A). The CPP scores were analysed with rm-ANOVA, using the TFF3 treatment (saline,0.01, 0.1 mg/kg) as the between-subjects factor and the test condition (Pre-T and Post-T) as the within-subjects factor. The behavioural results revealed no significant main effect for the TFF3 treatment [F(2, 18) = 0.192, p > 0.05] or test condition [F(1, 18) = 0.062, p > 0.05] and no TFF3 treatment × test condition interaction [F(2, 18) = 0.192, p > 0.05]. None of the groups of rats acquired conditioned place preference or aversion (p > 0.05, Fig. 6B), suggesting that TFF3 had neither an aversive nor rewarding effect per se.

## Discussion

The results of the present study showed that systemic TFF3 administration significantly augmented the cocaine-induced locomotor activity and cocaine-induced conditioned place preference. TFF3 administration significantly promoted cocaine-induced hyperactivity, enhanced the cocaine-induced increment of the dopamine concentration in the NAc and activity of mTOR signalling (as assessed by the analysed ratios of p-mTOR/mTOR and p-rpS6/rpS6) in the NAc shell but not the core. Moreover, the intra-NAc shell infusion of the mTOR inhibitor rapamycin blocked the hyperlocomotion activity induced by the prior TFF3 administration in cocaine-treated rats. Finally, systemic TFF3 administration had no reward or aversive effects *per se*. These findings suggest that mTOR signalling activation in the NAc shell is important for the regulatory effect of TFF3 on the cocaine-induced locomotor activity.

Neuropeptides have been identified to play important roles in modulating the rewarding value of abused drugs, as well as drug addiction^33^,^40^,^41^. Peng *et al*. reported that the microinjection of cocaine- and amphetamine-regulated transcript (CART) peptide into the NAc inhibited the cocaine-induced upregulation of dopamine receptors and locomotor sensitization^42^. Hypocretin (orexin) in the VTA enhanced cocaine-induced dopamine release and increased the cocaine seeking in self-administration rats model^43^,^44^. Chung *et al*. reported that a melanin-concentrated hormone system modulated the cocaine reward by potentiating the dopaminergic system in the NAc^45^. NPY decreased oral ethanol intake and yohimbine-induced reinstatement of alcohol seeking^46^,^47^, but increased the cocaine seeking behaviour in self-administration model and hyperlocomotion induced by acute cocaine injection^48^. Previous studies revealed that exogenous TFF3 administration (i.p.) modulated depression, anxiety, learning and memory^20^,^21^,^22^. In our previous studies we found that TFF3 level significantly increased in the BLA 30 min after systemic administration of TFF3, indicating indirectly that systemic TFF3 crosses the BBB^21^. Most recently, we reported that systemic TFF3 administration could attenuate naloxone-precipitated withdrawal in morphine-dependent mice^23^. Using the locomotor activity test and CPP rat paradigms, our present results showed that systemic TFF3 administration augmented the reward response to cocaine, highlighting the important role of TFF3 in drug addiction. In the current study, we found that pre-treatment of TFF3 enhanced the cocaine-induced hyperlocomotion and correspondingly increase in dopamine concentration in NAc. Acute administration of TFF3 alone had no effects on the dopamine concentration in the NAc could be interpreted by the modulatory effects of TFF3 on the release of dopamine or inhibition of DAT in the presence of cocaine. However, the specific mechanisms the underlying the TFF3 enhanced the cocaine-induced dopamine concentration in the NAc is still unknown. The possible mechanism refers to that systemic administration of TFF3 may has inhibitory effect on dopamine transporter in the NAc or enhance the function of dopamine neurons VTA, but the mechanisms need to be further studied.

Previous studies have shown that the TFFs can act on the EGF receptor, by which various downstream signalling pathways are activated, including the PI3K/PKB/Akt, MEK/ERK, MAPKs and mTOR signal pathways^13^,^24^. Accumulating evidence has suggested that the downstream signalling cascades of the TFFs, including the MEK/ERK, MAPKs and mTOR pathways, were also involved in the rewarding effects of abused drugs^49^,^50^,^51^,^52^,^53^. mTOR is an atypical serine/threonine kinase that responds to different extracellular stimuli to regulate cell growth or survival, transcription and protein synthesis^54^. Accumulating studies have demonstrated that mTOR is activated by the activation of NMDA and dopamine receptors^55^,^56^. Synaptic plasticity, which is regulated by mTOR, is required for fear and drug-related memory processes, including formation, consolidation, maintenance and reconsolidation^25^,^26^,^57^. The mTOR signalling pathway is also involved in drug-related behaviour, including cocaine or morphine-induced locomotor sensitization, as well as cue/drug-induced relapse in both human and animals^27^,^28^,^57^,^58^. In the present study, we found that TFF3 pre-treatment enhanced the increase in the phosphorylation of the mTOR signalling pathway in the NAc shell, but not the core, as well as the cocaine-induced locomotor activity and cocaine-induced conditioned place preference. Inhibition of mTOR activity in the NAc shell blocked the effect of TFF3 on cocaine-induced hyperlocomotion as well as corresponding changes in the mTOR signalling pathway, indicating that activity of the mTOR signalling pathway in the NAc shell is critically involved in the regulatory effect of TFF3 on the cocaine-induced locomotor activity and cocaine-induced conditioned place preference in rats. However, we found that rapamycin itself had no effect on cocaine-induced hyperactivity or rpS6 activity, but only inhibited TFF3-induced enhancement on cocaine-induced behavioral and molecular alterations, which may be caused by the dose effect of RAPA on inhibiting rpS6 activity demonstrated in our previous study^27^,^28^,^57^,^58^.

The NAc region is critical for mediating the rewarding effects of cocaine^9^,^28^,^59^,^60^. Our results found that acute cocaine administration significantly increased the dopamine concentration in the NAc and that systemic TFF3 administration augmented the increase in dopamine concentration in the NAc induced by acute cocaine exposure. This findings suggests that the modulatory effect of TFF3 on the behavioural response to cocaine is associated with the dopamine concentration, especially in the NAc region. As two subregions of the NAc region, the NAc core and shell have distinct immunohistochemical characteristics and afferent/efferent connections, which make the two brain regions dissociable heterogeneous structures that are differentially involved in drug addiction^61^,^62^. The NAc core plays an important role in the conditioned reinforcing effects of abused drugs-associated cues^28^,^63^,^64^,^65^, while the NAc shell mediates the rewarding effects of abused drugs because of the independent responses of the shell stimulated by excitatory amino acids and dopamine^61^,^66^. Therefore, the rewarding effects of a broad variety of physiological and pharmacological stimuli, such as abused drugs, are elicited from the NAc shell, but not the core^2^,^67^. Here, we found that TFF3 administration significantly enhanced the cocaine-induced changes in mTOR signalling activity in the NAc shell, but not the core. Furthermore, the inhibition of mTOR activity in the NAc shell blocked the TFF3-induced increase in hyperlocomotion activity in the cocaine-treated rats, providing further evidence for the different roles of the NAc shell and core in the cocaine-induced locomotor activity.

In summary, we demonstrated that TFF3 pre-treatment augmented cocaine-induced hyperlocomotion activity and conditioned place preference in rats. Furthermore, prior TFF3 administration enhanced the cocaine–induced increment of the DA concentration in the NAc and the activation of mTOR signalling in the NAc shell, but not the NAc core. Finally, the inhibition of mTOR signalling in the NAc shell, but not the core, blocked the effect of TFF3 on the cocaine-induced locomotor activity. These results, for the first time, revealed that TFF3 regulate the cocaine-induced locomotor activity and cocaine-induced conditioned place preference, which is associated with the dopamine concentration in the NAc and mTOR signalling activity in the NAc shell, but not core. These findings confirmed a new function of TFF3 in modulating cocaine-induced hyperlocomotion activity and cocaine-induced conditioned place preference and the underlying molecular mechanism.

## Concluding Remarks

Regulatory effects of neuropeptides is critical for cocaine cocaine-induced locomotor activity and cocaine-induced conditioned place preference. Our results indicate that neuropeptide Trefoil factor 3 (TFF3) potentiates the cocaine-induced locomotor activity and cocaine-induced conditioned place preference, which is associated with an enhanced increase in dopamine content within the NAc and the mTOR signalling in the NAc shell. The present findings, for the first time, provided direct evidence that TFF3 modulate the cocaine-induced locomotor activity and cocaine-induced conditioned place preference partially by activating mTOR signalling pathway in the NAc shell.

## Additional Information

**How to cite this article**: Luo, Y.-X. *et al*. mTOR signalling in the nucleus accumbens shell is critical for augmented effect of TFF3 on behavioural response to cocaine. *Sci. Rep*. **6**, 27895; doi: 10.1038/srep27895 (2016).
